# In-stent restenosis of left main chimney stent with successful percutaneous coronary intervention with drug-coating balloon: a case report and literature reviews

**DOI:** 10.1093/ehjcr/ytag093

**Published:** 2026-02-07

**Authors:** Yu Jui Hsieh, Chia-Pin Lin, Ying-Chang Tung, Fu-Chih Hsiao, Chi-Jen Chang

**Affiliations:** Division of Cardiology, Department of Internal Medicine, Chang Gung Memorial Hospital, No. 5 FuXing St., Guishan District, Taoyuan 33305, Taiwan; Division of Cardiology, Department of Internal Medicine, Chang Gung Memorial Hospital, No. 5 FuXing St., Guishan District, Taoyuan 33305, Taiwan; Division of Cardiology, Department of Internal Medicine, Chang Gung Memorial Hospital, No. 5 FuXing St., Guishan District, Taoyuan 33305, Taiwan; Division of Cardiology, Department of Internal Medicine, Chang Gung Memorial Hospital, No. 5 FuXing St., Guishan District, Taoyuan 33305, Taiwan; Division of Cardiology, Department of Internal Medicine, Chang Gung Memorial Hospital, No. 5 FuXing St., Guishan District, Taoyuan 33305, Taiwan

**Keywords:** TAVR, Chimney stent, In-stent restenosis, Cutting balloon, Drug coating balloon, Case report

## Abstract

**Background:**

Coronary artery obstruction (CAO) is a critical complication in transcatheter aortic valve replacement (TAVR). Coronary artery obstruction arises from mechanical obstruction of coronary ostia by displaced native or bioprosthetic valve leaflets. Despite the growing adoption of chimney stenting, data on long-term outcomes are sparse. We introduced a patient who had undergone a very late in-stent restenosis (ISR) over the left main (LM) chimney stent and its successful management using a cutting balloon (CB) and drug-coating balloon (DCB).

**Case summary:**

A patient experienced angina symptoms 2 years after receiving TAVR with LM chimney stent placement. Coronary angiography revealed ISR of LM chimney stent in neosinus proportion. The patient was successfully treated by CB and DCB angioplasty.

**Discussion:**

The chimney stent technique, initially developed as a bailout technique, is increasingly used prophylactically in anatomically high-risk cases. Lacking large-scale studies on chimney stent ISR limits our understanding. Besides CABG or repeat stenting, CB plus DCB may offer a potential opinion to achieve long-term benefits in such cases. With rapid increasing of TAVR patient, further studies may be needed to guide the possible complication with chimney stent in CAO cases.

Learning points

- The chimney stent technique can be used as a bailout or prophylactically in patient high-risk for coronary artery obstruction. However, the long-term outcomes remain questionable.
- Cutting balloon with drug coating balloon dilatation may be a potential treatment for in-stent restenosis of chimney stent.

## Introduction

The incidence of coronary artery obstruction (CAO) during native transcatheter aortic valve replacement (TAVR) is 0.6%, resulting in a 30-day mortality rate of 40%–50%.^1^ Chimney stent technique, extending the stent from proximal coronary artery cranially, is believed as an effective and safe approach for CAO.^2^ Despite its growing adoption, data on long-term outcomes, particularly stent durability and ISR risk, are sparse. We present a case of in-stent restenosis (ISR) over the left main (LM) chimney stent who had a successful percutaneous coronary intervention (PCI) using cutting balloon (CB) and drug-coating balloon (DCB), highlighting the potential risk of stent failure of chimney stent and the benefit of CB and DCB in treatment.

## Summary figure

**Figure:**
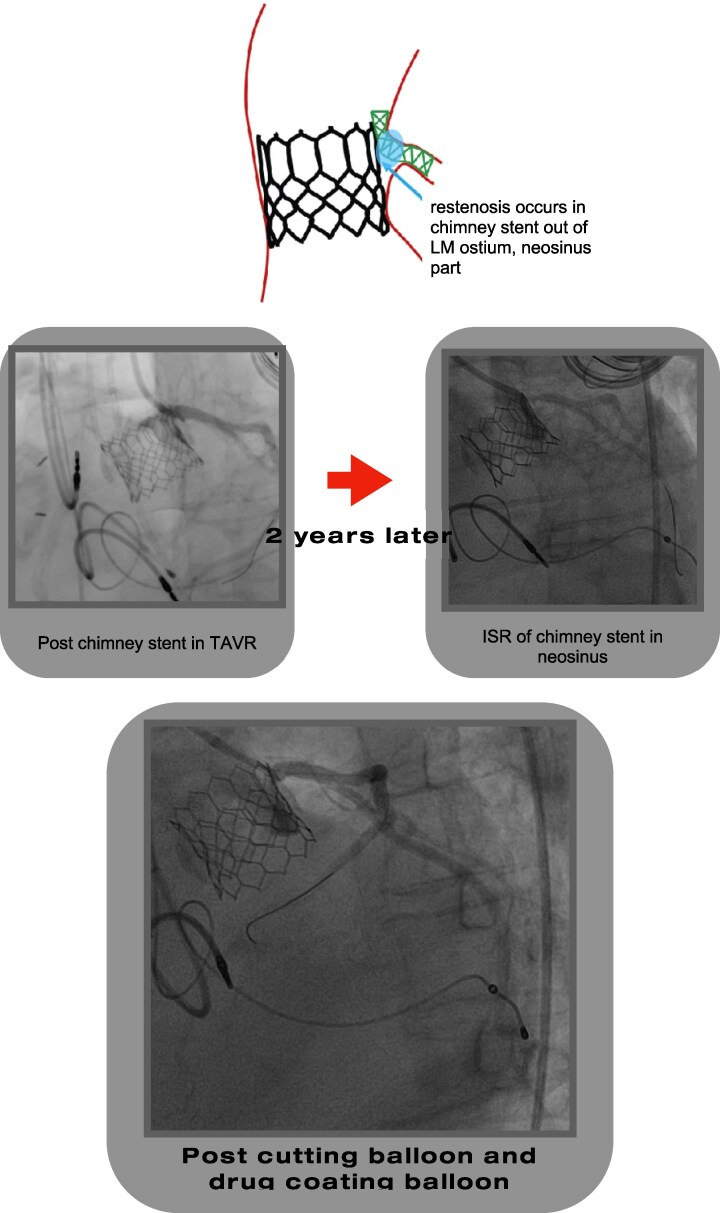
Late ISR in a chimney stent in neosinus with successful treatment by CB and DCB. LM, left main; TAVR, transcatheter aortic valve replacement; ISR, in-stent restenosis.

## Case presentation

A 65-year-old woman presented with progressive dyspnoea on exertion over the past months, ultimately limiting her ability to walk more than 100 m. She had a known history of ischaemic cardiomyopathy with heart failure with mildly reduced ejection fraction (HFmrEF with LVEF 42%), hypertension, diabetes mellitus, chronic kidney disease, and a prior history of malignancy in remission. Eight years earlier, she had received radiotherapy to the right lung field. Her medications included apixaban, losartan, bisoprolol, nicorandil, furosemide, empagliflozin-linagliptin combination, and ezetimibe-simvastatin.

**Figure 1:**
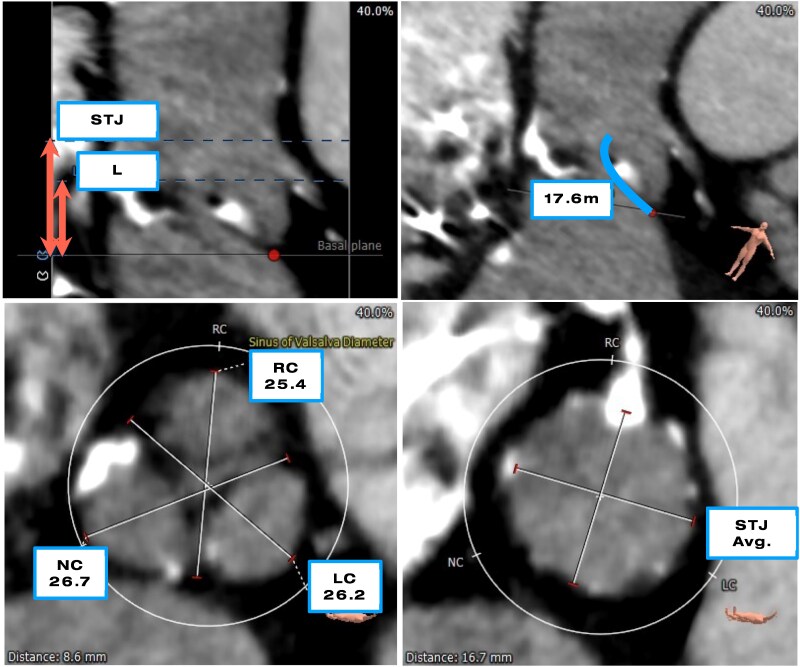
Pre-procedural planning with multi-slice computed tomography demonstrating considerable risk of left coronary artery obstruction with low coronary ostium height, 11.0 mm of left coronary artery (*A*). Calcified left coronary leaflet and leaflet length 17.6 mm (*B*) and shallow sinuses of Valsalva with diameter 26.2 mm at left cuspid (*C*). Sinotubular junction 22.3 mm in average diameter (*D*).

Her cardiac history was notable for prior RCA occlusion treated with successful PCI and drug-eluting stent (DES) implantation. She also had a cardiac resynchronization therapy pacemaker (CRT-P) for HFrEF with complete atrioventricular block. This improved her LVEF to 56% in 2020 in the presence of moderate aortic stenosis (AS). In May 2022, she was diagnosed with severe AS and a declining LVEF. She underwent TAVR with a 23 mm Edwards Sapien 3 valve. Pre-procedural computed tomography revealed a high CAO risk, with a shallow sinus of Valsalva 25.4 mm in mean diameter and a low coronary height 11.0 mm at left coronary artery (LCA) (*Figure 1*).

**Figure 2:**
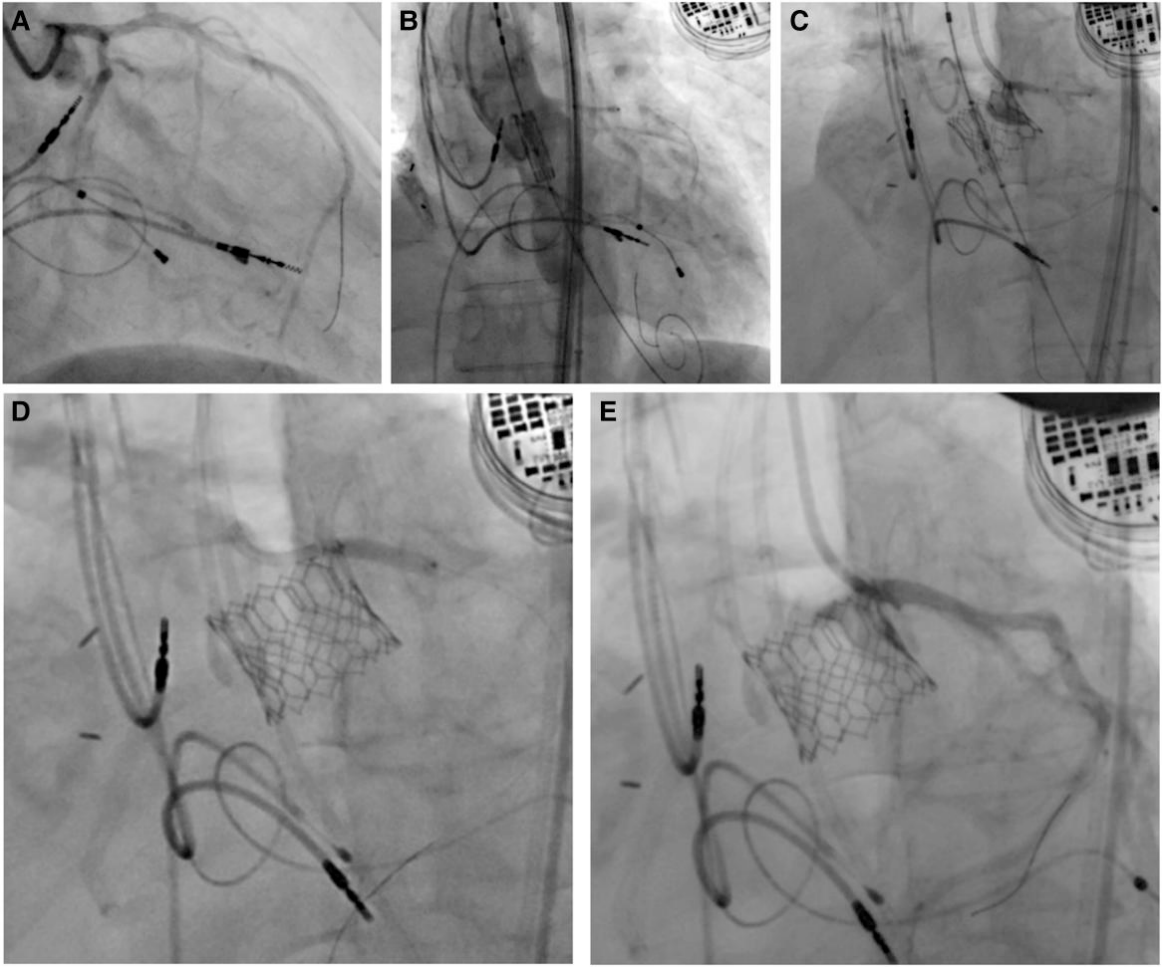
Coronary ostium obstruction in transcatheter aortic valve replacement. Patent ostial left main before transcatheter heart valve deployment (*A*). Pre-positional stent at left main before transcatheter heart valve deployment (*B*). Ostial left main partial obstruction by native valve after transcatheter heart valve placement (*C*). Increasing ostial left main diameter after chimney stent deployment and post-dilatation (*D* and *E*).

Left coronary artery was protected with a stent preposition. As the orifice of LCA was found partially compromised by native valve leaflet after transcatheter heart valve (THV) implantation, a DES (Abbott Xience 3.5 × 15 mm) was deployed at LM in chimney technique (*Figure 2*), which successfully deformed the native valvular tissue. The patient was discharged uneventfully and remained asymptomatic over 2 years.

In July 2024, she developed exertional dyspnoea, prompting re-evaluation. Transthoracic echocardiogram showed a well-functioning aortic valve prosthesis with a mean gradient of 11 mmHg, estimated valve area 1.5 cm^2^ by Doppler method, and preserved left ventricular ejection fraction of 59%.

Coronary angiography was performed (*Figure 4*), revealing severe ISR of the LM chimney stent (Video S1). The RCA appeared to be in-stent occluded with poor visualization (non-dominant). Selective engagement of the LM was technically challenging due to its position within the stented segment and the THV.

**Figure 3:**
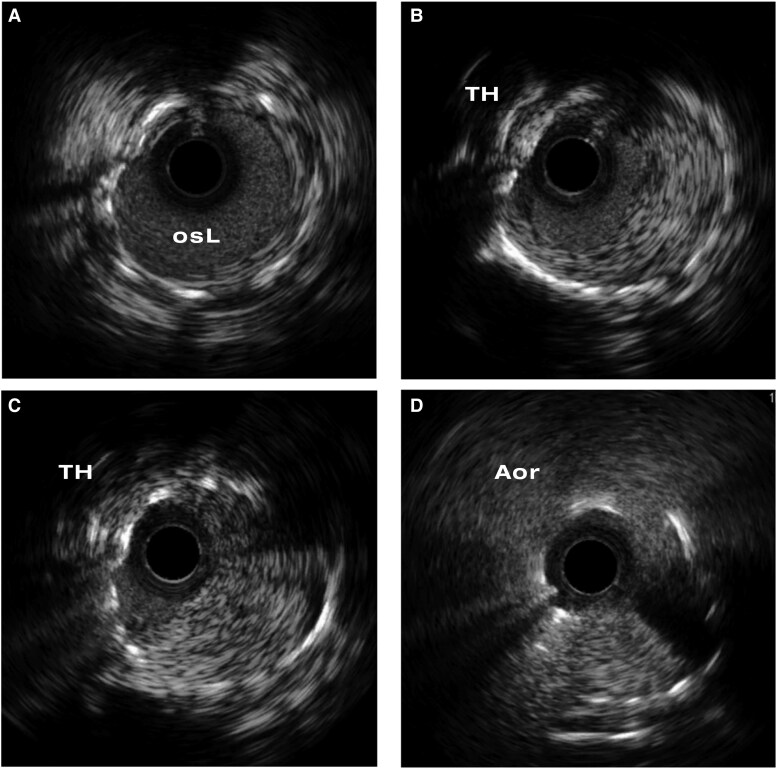
Intravascular ultrasound of chimney stent in-stent restenosis. Patent stent inside of left main and ostium (*A*). Neointima formation at chimney stent out of left main with contralateral compressive deformity by transcatheter heart valve stent (*B* and *C*). No stenosis at the ostium of chimney stent (*D*).

Using a left radial approach, a 6 Fr. EBU3.5 guiding catheter was manoeuvred through the THV cell to reach the chimney stent. A Fielder FC guidewire (Asahi) was advanced into distal LAD using a fishing technique. Intravascular ultrasound (IVUS) confirmed that the wire accessed the true lumen of chimney stent and revealed the ISR lesion with homogenous neointimal tissue at the proximal portion of the stent hanging in the proportion external to the LM artery (*Figure 3*, Video S2).

**Figure 4:**
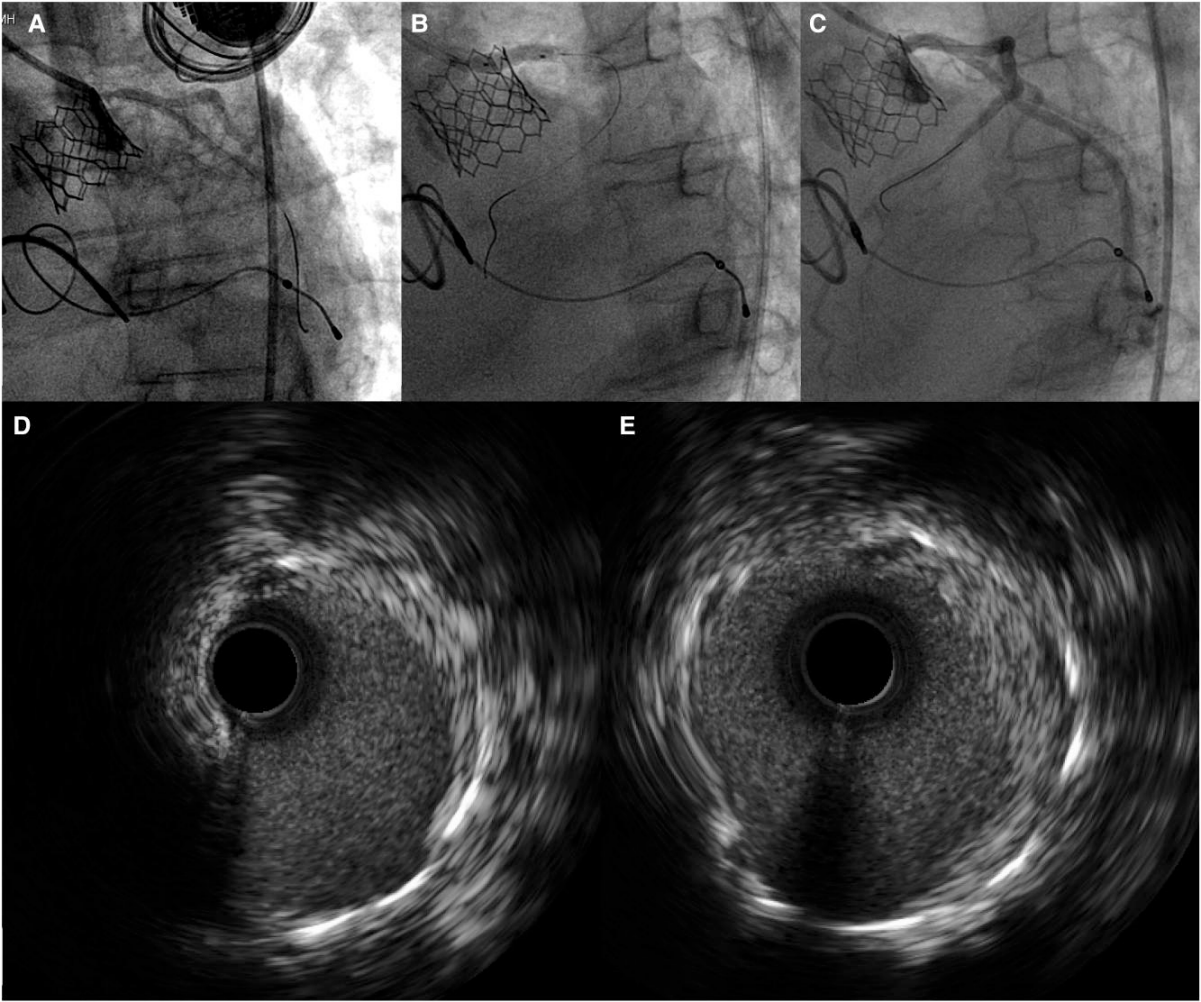
In-stent restenosis percutaneous coronary intervention to the left main chimney stent. In-stent restenosis of chimney stent inside neosinus (*A*). Post POBA, including cutting balloon dilatation and drug-coating balloon over chimney stent at the left main (*B*). Regain the potency of chimney stent in left main in final angiogram (*C*). The intravascular ultrasound image of stenosis of chimney stent post CB and DCB dilatation (*D* and *E*) with image (*D*) position correlating to *Figure 3B* and image (*E*) to *Figure 3C*.

Lesion preparation was performed using 3.0 and 4.0 mm non-compliance balloons, followed by a 4.0 mm CB. Subsequently, a 4.0 mm DCB (Biotronik Pantera Lux) was inflated at the ISR site (Vedio S3). Final IVUS image showed satisfactory stent expansion with a minimal luminal area (MLA) of 9.8 mm^2^ (*Figure 4*, Video S4). The patient was discharged without complications and remained symptom-free during 1 year of follow-up.

## Discussion

The chimney technique involves placing a stent in the coronary ostium, extending into the aorta, to maintain blood flow when there is a risk of CAO post TAVI.^2,3^ The stent could be parked prophylactically in coronary artery before deployment of THV in high-risk patients identified by pre-procedural imaging or delivered immediately after TAVR when acute CAO occurs.^1,2^ Despite its utility, the technique has limitations including technical challenges with precise stent placement and apposition, risk of stent thrombosis, potential migration, and increased procedure complexity. Our case underscores a late complication—ISR—in a chimney stent (localized mainly) to the segment overhanging in the sinus of Valsalva, which is rarely reported but clinically significant, particularly when it occurs in LM coronary artery.

Two cases of ISR following chimney stenting for RCA have ever been reported. Both cases present with recurrent angina or shortness of breath months to years after the initial procedure.^2,3^ One was eventually treated by CABG^2,4^ and the other one by PCI with another DES deployment.^3^ In the case we reported, ISR occurred in chimney stent implanted for LCA with partial CAO. To our knowledge, this is the first case that ISR was treated using CB along with DCB without additional stenting, which result in an acute procedural success and a favourable mid-term clinical outcome.

The lack of large-scale, dedicated studies on chimney stent ISR limits our understanding of the mechanism of ISR in this unique stenting fashion. In the chimney stent, the overhanging stent segment protruding into the partially sequestrated sinus of Valsalva is exposed to an unfavourable haemodynamic condition. This segment is only partially supported by surrounding tissue and was exposed to stagnated flow with reduced flow velocity and low shear stress.^5^ This can increase the risk of thrombosis and neointimal hyperplasia even with the stent fully expanded.^6^ In the case presented, intravascular imaging with IVUS demonstrated well-stent expansion and neointimal tissue growth primarily in the overhanging segment.

Interestingly, the overhanging segment of the stent has evolved from a slotted tube to a hollow tube supported by neointimal tissue. This is a result of successful CB dilation which creates a widely opened lumen. These morphological changes may facilitate a laminar flow rather than a disturbed flow within the stent. This favourable flow pattern, in conjunction with the effect of DCB,^7–9^ may help to maintain stent patency.

In-stent restenosis remains a significant challenge following chimney stenting. While DCBs offer a potential treatment avenue, further research is required to determine their efficacy in this specific context. A better understanding of the underlying mechanisms will guide the development of preventative and therapeutic strategies ultimately improving patient outcomes.

Intravascular imaging was crucial in differentiating the aetiology in this case. In contrast to OCT, IVUS is preferable in such instances.^10^ The placement of a chimney stent would extend from the LM vessel into the neosinus making OCT difficult to assess the stent characteristics from coronary vessels. Particularly for the lesion in the neosinus as in our case, IVUS may provide a more effective tool for evaluation.

In this patient, LVEF showed dynamic changes over time. Following CRT, LVEF initially improved to 56% when only moderate AS was present. However, in the subsequent year, severe AS was first diagnosed, and by the following year, LVEF had declined to 45%. After TAVR, LVEF improved again. We believe the observed LVEF deterioration prior to TAVR was primarily driven by the progression to severe AS, which imposed significant afterload on an already compromised left ventricle in the setting of underlying ischaemic cardiomyopathy. Relief of this afterload through TAVR likely contributed to the subsequent LVEF recovery.

In patients with CAO post TAVI, chimney stent could be a lifesaving procedure.^11^ However, it is controversial to perform chimney stent for patients with partial CAO. In our patient, a DES was deployed in partially compromised LM after TAVR, meeting with stent failure 2 years later. Further study is mandatory to guide the treatment for patients with partial CAO post TAVI.

## Lead author biography


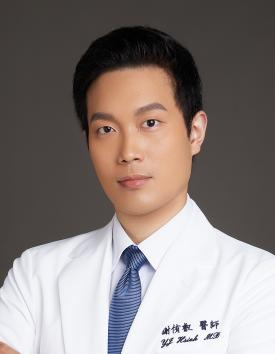


Dr Yu Jui Hsieh completed his specialty in internal medicine and subspecialty in cardiology at Linkou Chang Gung Memorial Hospital in Taiwan and is currently an interventional cardiologist in the same institution.

## Supplementary Material

ytag093_Supplementary_Data

## Notes

**Consent:** The study was conducted with the Declaration of Helsinki and approved by the Institutional Review Board of Chang Gung Medical Foundation (protocol code 202500392B0; approved in Jul. 2025). Informed consent was obtained from all subjects involved in the study. The authors confirm that written consent for submission and publication of this case report including images and associated text has been obtained from the patient, in line with COPE guidance.
