# Supplementary material for: In-stent restenosis of left main chimney stent with successful percutaneous coronary intervention with drug-coating balloon: a case report and literature reviews
Source: Eur Heart J Case Rep. 2026 Feb 7;10(3):ytag093. doi: 10.1093/ehjcr/ytag093 (PMC12952206; doi:10.1093/ehjcr/ytag093)
Supplement: ytag093_Supplementary_Data [file ytag093_supplementary_data.zip › FigureS1_Timeline.docx]

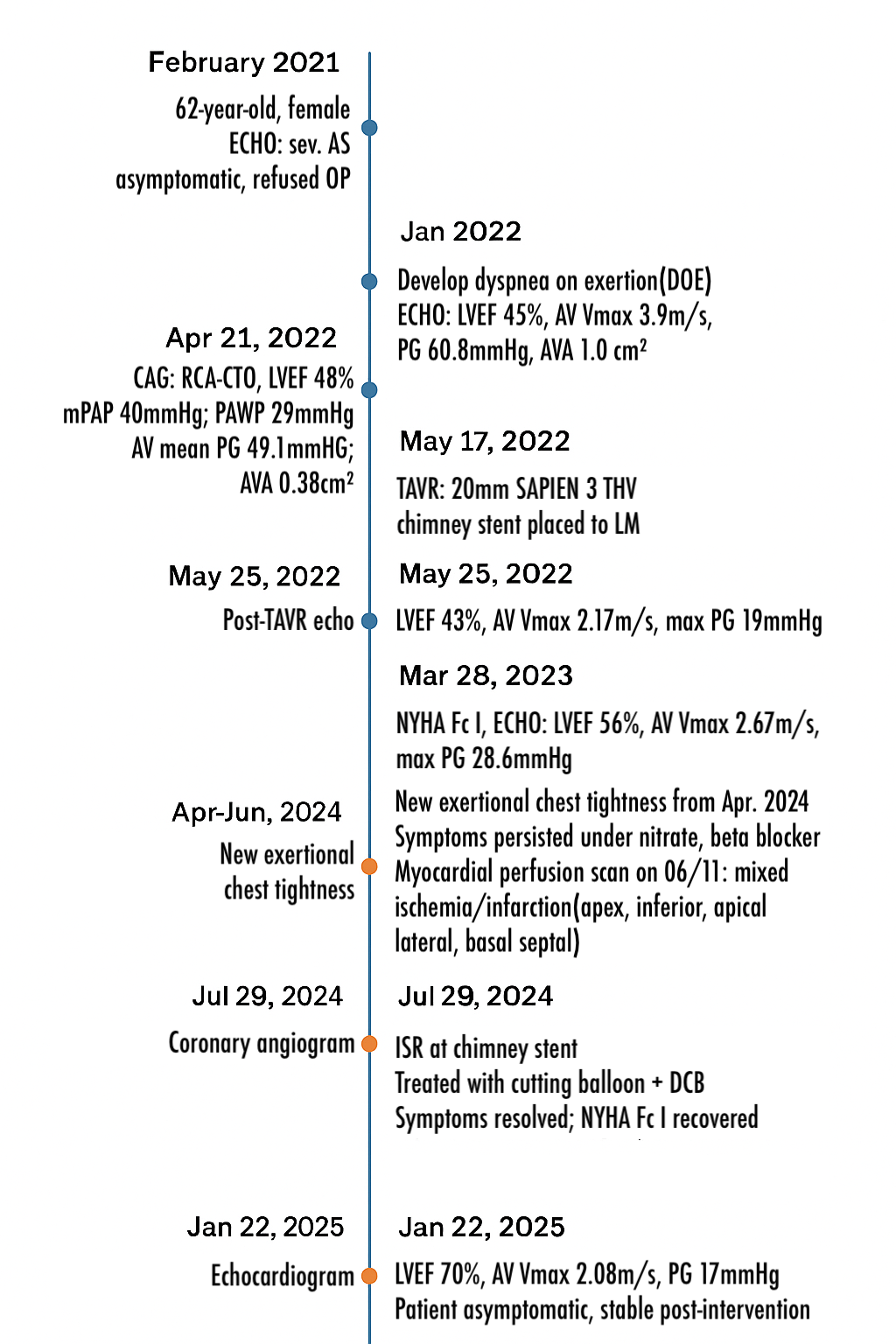


**Figure S1.** Clinical timeline of a 62-year-old female patient with severe aortic stenosis managed with transcatheter aortic valve replacement (TAVR).
